# Assessment of brain penetration and tumor accumulation of niraparib and olaparib: insights from multimodal imaging in preclinical models

**DOI:** 10.1038/s41598-025-18255-9

**Published:** 2025-09-29

**Authors:** M. Reid Groseclose, Jeremy A. Barry, Tina Skedzielewski, Yongle Pang, Yinghe Li, Gerald McDermott, Jennifer Deutsch, Chakravarthi Balabhadrapatruni, William Benson, David Lim, Hoang Tran, Elisabeth Minthorn, Casey Kmett, Theresa Roethke, Shannon Berry, Elaina McCormick, William Feeney, M. A. Ringenberg, Sean Maguire, Geeta Sharma, Amine Aziez, Elaine M. Paul, Kunal Taskar, Keyur Gada, Hasan Alsaid

**Affiliations:** 1 Bioimaging, GSK, Collegeville, PA USA; 2 DMPK, GSK, Collegeville, PA USA; 3 Laboratory Animal Sciences and Governance, GSK, Collegeville, PA USA; 4 Oncology Research Unit, GSK, Collegeville, PA USA; 5 Research Statistics, Biostatistics, GSK, Collegeville, PA USA; 6 Nonclinical Safety, GSK, Collegeville, PA USA; 7 Global Medical Affairs Oncology, GSK, Zug, Switzerland; 8 Oncology Medicine Development, GSK, Collegeville, PA USA

**Keywords:** Drug delivery, Cancer imaging, Cancer models, Pharmacokinetics, Imaging studies, Mass spectrometry

## Abstract

One of the most significant challenges in developing new treatments for primary and metastatic brain tumors is the requirement for crossing the blood-brain barrier (BBB). By examining the brain penetration and spatial distribution of drug candidates in animal models, we can gain valuable insights into their potential for clinical success. In this study, we used multimodal imaging alongside complementary analytical methods to assess the brain distribution of two clinically advanced poly(ADP-ribose) polymerase (PARP) inhibitors—niraparib and olaparib—across three preclinical models: healthy brain (nonhuman primates (NHPs)), primary brain tumors (mouse glioblastoma), and metastatic brain tumors (mouse breast cancer metastasis). The results suggest that niraparib has a superior ability (relative to olaparib) to penetrate the BBB, distribute widely throughout the brain, and accumulate in brain tumor lesions. These findings support further studies of niraparib as a potential treatment for tumors in the human brain.

**Supplementary Information:**

The online version contains supplementary material available at 10.1038/s41598-025-18255-9.

## Introduction

Systemic treatment options for primary and metastatic brain tumors remain limited compared with those available for extracranial cancers. Survival outcomes in patients with glioblastoma - the most common primary malignant brain tumor - are poor, with a 5-year relative survival rate of only 6.8%^1^. Prognoses for patients with metastatic brain tumors are similarly poor^2^, underscoring a significant unmet need for new treatment options. One major obstacle is the inability of many systemic treatments to cross the blood–brain barrier (BBB) and effectively reach tumors in the central nervous system (CNS). The BBB allows for passive diffusion of small, lipid-soluble drugs but sharply restricts paracellular diffusion of polar solutes to only uptake substrates of carrier-mediated transporters^3^. Furthermore, active efflux transporters, such as P-glycoprotein (ABCB1/MDR1) and breast cancer resistance protein (ABCG2), transport many molecules out of the CNS that normally diffuse across cell membranes^4,5^.

To assess brain drug distribution, the unbound brain-to-plasma partition coefficient (K_p, uu, brain_), has become the industry standard to describe the pharmacokinetics of CNS-targeted drugs during discovery and development^6,7^. First introduced in 2006^8^, K_p, uu, brain_ describes the unbound drug concentration in the brain compared with that in blood plasma and reflects the net balance of drug influx and efflux across the BBB^6,7^.

Poly(ADP-ribose) polymerase (PARP) is a stress sensor that plays a key role in DNA repair by responding to damage including single-strand breaks (SSBs) and activating the DNA repair machinery. In tumor cells with defects in the homologous recombination DNA repair pathway – most notably those with BRCA1 or BRCA2 mutations – inhibition/disruption of PARP-mediated repair of SSBs results in the accumulation of DNA damage and ultimately cancer cell death^9^. This synergistic disruption of two gene products resulting in cell death, whereas disruption of only one maintains cell viability, is called synthetic lethality. Discovery of this synthetic lethal interaction between PARP inhibition and BRCA1/2 mutations has led to the development of several PARP inhibitor drugs designed to exploit this pathway for anticancer therapy^10–12^. Niraparib is a PARP inhibitor approved as a maintenance therapy for recurrent ovarian, fallopian tube, or primary peritoneal cancer that has responded to platinum-based chemotherapy^13,14^ and as part of a combination therapy for patients with *BRCA*-mutant metastatic castration-resistant prostate cancer^15^. Olaparib, another small-molecule PARP inhibitor, is approved for use in patients with *BRCA-*mutant advanced ovarian cancer^16^, in patients with *BRCA*-mutant breast cancer^17^, in patients with *BRCA*-mutant metastatic pancreatic adenocarcinoma, and in patients with homologous recombination repair–mutant metastatic castration-resistant prostate cancer^18^. Currently, no PARP inhibitors are approved to treat CNS tumors, but there is considerable interest in exploring their utility in brain cancers like glioblastoma, given their ability to augment DNA damage induced by radiotherapy and chemotherapy^19,20^.

PARP inhibitors such as rucaparib and olaparib have been shown to have poor penetration across the BBB^21,22^. However, olaparib was detected in the tumor tissue of an orthotopic glioblastoma xenograft model with a disrupted BBB, suggesting that a partially compromised BBB can permit some drug distribution to the tumor site^22^. Niraparib, by contrast, has been associated with increased brain exposure relative to other PARP inhibitors in preclinical models including an intracranial tumor model using a pancreatic cancer cell line^23^. Recently, pharmacologically relevant concentrations of niraparib have been observed in brain tumor tissue from patients with newly diagnosed glioblastoma^24–27^.

In this study, we investigated the brain penetration and spatial distribution of niraparib and olaparib in three preclinical settings: healthy nonhuman primates (NHPs), a mouse model of glioblastoma, and a mouse model of metastatic brain tumors (using a human breast cancer cell line). We employed multimodal imaging and quantitative analysis to compare the extent of BBB penetration and tumor accumulation for the two PARP inhibitors across these models.

## Results

### Brain penetration and distribution in healthy rhesus macaques

The LC-MS/MS bioanalysis results for plasma, CSF, and brain homogenates from healthy NHPs administered niraparib or olaparib are summarized in Table 1. In brain homogenates, substantially greater brain penetration was observed in the two NHPs dosed with niraparib (378 and 797 ng/g; mean K_p, brain_, 3.179) than in the two NHPs dosed with olaparib (12 and 11 ng/g; mean K_p, brain_, 0.041). The mean K_p, uu, brain_ (unbound brain-to-plasma ratio) was approximately 12-fold higher for niraparib (0.313) than olaparib (0.026). It should be noted that the cerebral blood volume is ~ 3–4% of the total brain homogenate; thus, a K_p, brain_ value of ~ 0.04 or lower primarily reflects drug content in the brain vasculature rather than true parenchymal penetration^28^. The mean K_p, brain_ for olaparib was estimated at ~ 0.041, suggesting that olaparib’s measured brain levels in these NHPs largely represent cerebral blood-associated drug. Accordingly, the actual K_p, brain_ and K_p, uu, brain_ ratios for niraparib versus olaparib are likely even higher than the nominal values reported above.

**Table 1:** Drug concentrations measured by MALDI MSI and LC-MS/MS bioanalysis in healthy rhesus macaque NHPs after oral administration of niraparib (6 mg/kg) or Olaparib (10 mg/kg) once daily for 5 Days.

	Animal	MALDI MSI^a^	LC-MS/MS bioanalysis^b^			K_*p*, brain_^e^	Mean K_*p*, brain_	K_*p*, uu, brain_^f^	Mean K_*p*, uu, brain_
		Brain sections, mean (SD), ng/g^c^	Terminal* plasma, ng/mL^c^	Terminal* CSF, ng/mL^c^	Bulk brain tissue, ng/g^c^				
Niraparib	NHP 1	283 (69)	84	9	378	4.530	3.179	0.446	0.313
	NHP 2	684 (189)	436	25	797	1.829		0.180	
Olaparib	NHP 3	< LOD	322	13	12	0.037	0.041	0.024	0.026
	NHP 4	< LOD	254	6	11	0.044		0.028	

*CSF* cerebrospinal fluid, *C*_*brain,ss*_ steady-state total drug concentration in bulk brain tissue, *C*_*plasma,ss*_ steady-state (terminal) total drug concentration in plasma, *C*_*u,brain,ss*_ steady-state unbound drug concentration in brain interstitial fluid, *C*_*u,plasma,ss*_ steady-state unbound drug concentration in plasma, *K*_*p,brain*_ brain-to-plasma partition coefficient, *K*_*p,uu,brain*_ unbound brain-to-plasma partition coefficient, *LC-MS/MS* liquid chromatography–tandem mass spectrometry, *LLOQ* lower limit of quantification, *LOD* limit of detection, *MALDI MSI* matrix-assisted laser desorption/ionization mass spectrometry imaging.

^a^The MALDI MSI LOD was 70 ng/g for olaparib and 20 ng/g for niraparib.

^b^LC-MS/MS bioanalysis LLOQ was 2 ng/mL for niraparib in all matrices and was 1 ng/mL for olaparib in all matrices.

^c^Values are total (unbound + bound) concentrations.

^d^Values are the mean of the mean concentration measured from each tissue section.

^e^Calculated as ratio of C_brain, ss_ versus C_plasma, ss_.

^f^Calculated as ratio of C_u, brain, ss_ versus C_u, plasma, ss_.

*Terminal plasma and terminal CSF are post-mortem samples collected during necropsy.

**Fig. 1:**
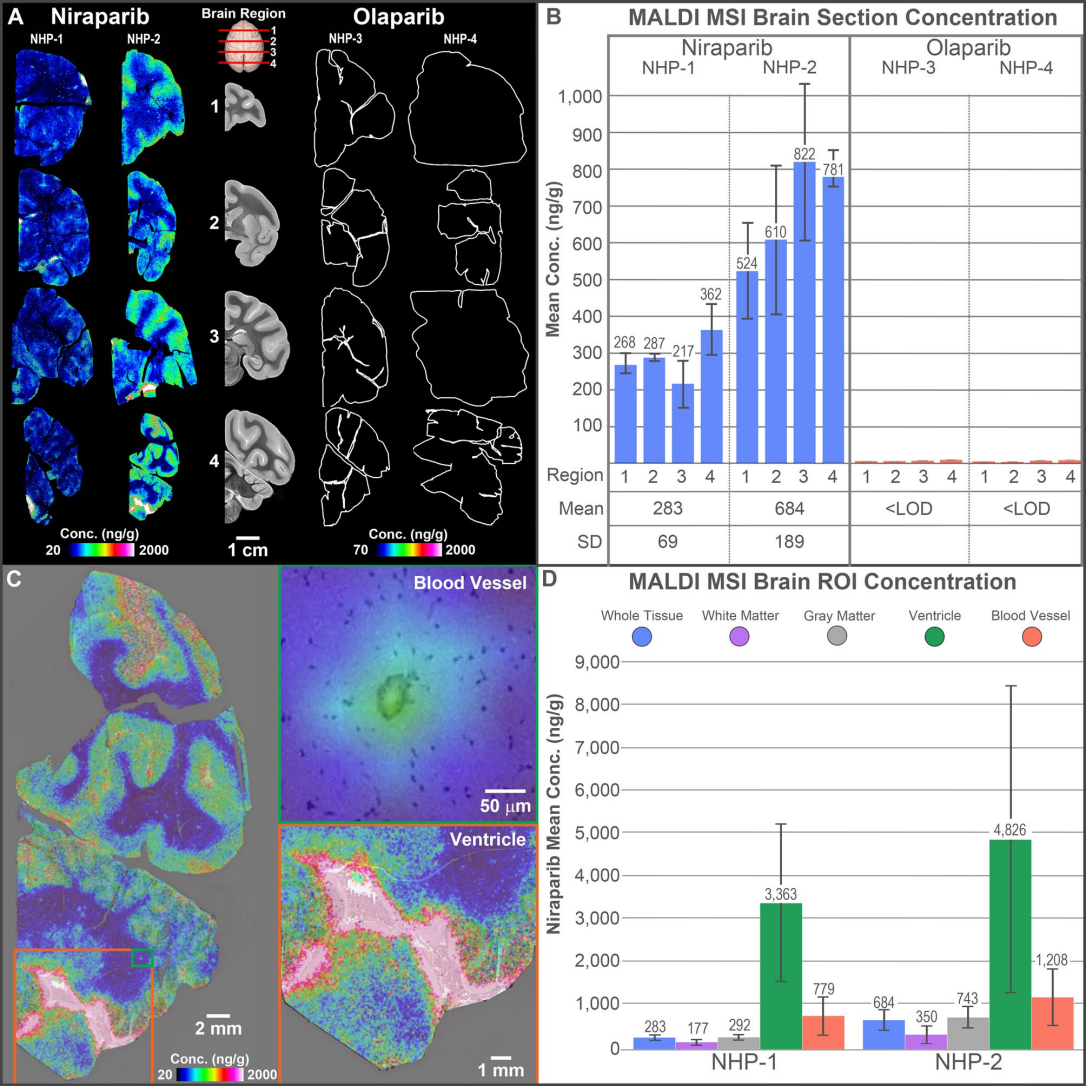
Brain penetration in healthy rhesus macaques. (**A**) Quantitative MALDI MSI images acquired from representative brain tissue sections collected at four different anatomical planes from NHPs administered either niraparib or olaparib. The center panel (grey images labelled 1–4) shows an approximate anatomical brain location where each section was collected. White outlines are drawn to indicate the tissue boundaries in sections where no drug signal was detected (for visualization purposes). (**B**) Above: Mean concentration bar plots (SD error bars) for tissue sections from each region (3 sections for each region) for each NHP administered either niraparib or olaparib. Below: Table of the overall mean brain concentration and standard deviation for all sections (12 per animal) for each respective animal. (**C**) Representative brain tissue section from an NHP administered niraparib displayed as an overlay of the niraparib ion image generated by MALDI MSI with an H&E-stained section, and magnified views of a blood vessel and ventricle region. (**D**) Bar plots displaying the mean concentration and standard deviation (error bars) for niraparib in the annotated histological ROIs across all sections for each NHP. The estimated limit of detection by MALDI MSI is 20 ng/g for niraparib and 70 ng/g for olaparib. * Conc.* concentration, *H&E* hematoxylin and eosin, *LOD* limit of detection, *MALDI MSI* matrix-assisted laser desorption/ionization mass spectrometry imaging, *ROI* region of interest.

Plasma and CSF concentrations were similar between niraparib-dosed NHPs (84 and 436 ng/mL in plasma; 9 and 25 ng/mL in CSF) and olaparib-dosed NHPs (322 and 254 ng/mL in plasma; 13 and 6 ng/mL in CSF). Quantitative MALDI MSI analysis of coronal brain tissue sections revealed niraparib distributed across all examined brain regions, whereas olaparib was not detected (i.e. < LOD) in any of the examined brain sections (Fig. 1A and B). The observed difference in concentrations between niraparib and olaparib was substantial; however, given the limited number of animals (*N* = 2 per group) this result should be interpreted cautiously as an indicative trend rather than a definitive statistical finding. The niraparib brain concentrations estimated by MALDI MSI spatial quantification were consistent with the bulk brain tissue concentrations measured by LC-MS/MS bioanalysis for the niraparib dosed NHPs. The concentrations of olaparib measured in the brain homogenates by LC-MS/MS (12 and 11 ng/g) were below the LOD of olaparib by MALDI MSI (~ 70 ng/g under our experimental conditions). The LOD of MALDI MSI is higher on a nanogram-per-gram basis than LC-MS/MS because of the very small quantity of tissue sampled at each pixel with imaging and the inability to concentrate the sample. Overlaying the niraparib ion images onto serial H&E-stained brain sections revealed that the localized concentration of niraparib was higher in the ventricles than in the blood vessels and brain parenchyma (Fig. 1C and D).

Given the localized high concentrations of niraparib measured in the ventricle, it would have been expected that high concentrations were also measured in the terminal CSF; however, the CSF concentrations were low (Table 1). Further review of the niraparib ion images generated by MALDI MSI co-registered with serial H&E stained tissues, revealed that niraparib is highly localized to the choroid plexus cells within the ventricles and not more generally distributed throughout the ventricles, as shown in Figure S2. The significance of this observation or the mechanism underlying it is not known.

### Brain penetration in a mouse model of brain metastasis

The brain penetration and tumor distribution of niraparib and olaparib were also evaluated in the mouse metastatic tumor model. After intracardiac (IC) injection of MDA231-BrM2-831 cells, the formation of brain metastases (BM) was confirmed by bioluminescence imaging (BLI) (Figure S1A and S1B). IHC staining for human cytochrome c oxidase subunit 4 (COXIV) confirmed the non-mouse origin of cells constituting the cerebral tumors in this model (Figure S1C and S1D). Histopathological review only found brain tumors in mice that had a correspondingly high BLI signal; no tumors were observed in the brains of mice lacking a BLI signal.

LC-MS/MS and MALDI MSI results for this study are summarized in Table 2. Analysis of bulk brain homogenates by LC-MS/MS showed that niraparib concentrations were higher than olaparib concentrations in both the brains of no-BM mice (niraparib 658 ng/g vs. olaparib 5 ng/g) and BM mice (niraparib 543 ng/g vs. olaparib 6 ng/g). For both niraparib and olaparib dosing groups, the brain concentrations for the BM and no-BM mice were similar (niraparib: 658 ng/g no-BM vs. 543 ng/g BM; olaparib: 5 ng/g no-BM vs. 6 ng/g BM), indicating that the presence of brain tumors did not significantly affect the overall brain drug levels in this model. The plasma concentrations of niraparib (3847 ng/mL no-BM; 2535 ng/mL BM) were more than tenfold higher than those of olaparib (226 ng/mL no-BM; 119 ng/mL BM), consistent with previous studies showing that olaparib’s oral bioavailability is lower than that of niraparib, even when olaparib is administered at a higher dose^23^. Across all mice (BM and no-BM combined), the mean K_p, brain_ for niraparib (0.193) was approximately fivefold higher than the K_p, brain_ for olaparib (0.036). The calculated K_p, uu, brain_ ratio (niraparib vs. olaparib) was ~ 1.81 in no-BM mice and 0.93 in BM mice, giving an overall mean K_p, uu, brain_ only about 1.2-fold higher for niraparib (0.026) than olaparib (0.022). However, similar to the primate study, the mean olaparib K_p, brain_ in mice was around ~ 0.036, therefore the olaparib brain concentration likely reflects mostly residual cerebral blood content. Thus, the actual K_p, brain_ and K_p, uu, brain_ ratios for niraparib versus olaparib are probably greater than the nominal K_p, uu, brain_ ratios suggest. The concentrations of both niraparib and olaparib were below the LOD in both the plasma and brains of vehicle-dosed mice by both MALDI MSI and LC-MS/MS (Table 2).

**Table 2:** Drug concentrations measured by MALDI MSI and LC-MS/MS bioanalysis in a mouse model of BM after oral administration of niraparib (35 mg/kg) or Olaparib (50 mg/kg) once daily for 5 Days.

		MALDI MSI^a^		LC-MS/MS^b^		Group mean (SD) K_*p*, brain_	Mean K_*p*, brain_	Group mean (SD) K_*p*, uu, brain_^d^	Mean K_*p*, uu, brain_	K_*p*, tumor_^e^
		Brain sections, mean (SD), ng/g^c^	Brain tumors, mean (SD), ng/g^c^	Terminal* plasma, mean (SD), ng/mL^c^	Bulk brain tissue, mean (SD), ng/g^c^					
Niraparib	No BM (*n* = 3)	519 (194)	–	3847 (595)	658 (71)	0.171 (0.023)	0.193	0.024 (0.003)	0.026	–
	BM (*n* = 4)	487 (200)	2396 (2155)	2535 (414)	543 (27)	0.214 (0.032)		0.030 (0.004)		0.945
Vehicle^f^	Control (*n* = 3)	< LOD	–	BQL	BQL	–	–	–	–	–
Olaparib	No BM (*n* = 3)	< LOD	–	226 (75)	5 (4)	0.027 (0.001)	0.036	0.015 (0.004)	0.022	–
	BM (*n* = 3)	< LOD	< LOD	119 (63)	6 (5)	0.102 (0.081)		0.047 (0.046)		–
Vehicle^g^	Control (*n* = 3)	< LOD	–	BQL	BQL	–	–	–	–	–

BM, brain metastasis; BQL, below quantification limit; C_u, brain, ss_, steady-state unbound drug concentration in brain interstitial fluid; C_u, plasma, ss_, steady-state unbound drug concentration in plasma; F_u_, fraction of unbound drug; K_p, brain_, brain-to-plasma partition coefficient; K_p, tumor,_ tumor-to-plasma partition coefficient; K_p, uu, brain_, unbound brain-to-plasma partition coefficient; LC-MS/MS, liquid chromatography–tandem mass spectrometry; LLOQ, lower limit of quantification; LOD, limit of detection; MALDI MSI, matrix-assisted laser desorption/ionization mass spectrometry imaging; ROI, region of interest.

^a^MALDI MSI LOD was 170 ng/g for olaparib and 70 ng/g for niraparib.

^b^LC-MS/MS bioanalysis LLOQ was 2 ng/mL for niraparib in all matrices and 1 ng/mL for olaparib in all matrices.

^c^Values are total (unbound + bound) concentrations.

^d^Calculated as ratio of C_u, brain, ss_ (mean total brain tissue concentration adjusted for F_u_) versus C_u, plasma, ss_ (terminal total plasma concentration adjusted for F_u_).

^e^Calculated as ratio of mean brain tumor ROI total concentration measured by MALDI MSI versus terminal plasma total concentration.

^f^Brain tissue from each of the three vehicle-treated mice were evaluated for niraparib.

^g^Brain tissue from each of the three vehicle-treated mice were evaluated for olaparib.

*Terminal plasma is a post-mortem sample collected during necropsy.

**Fig. 2:**
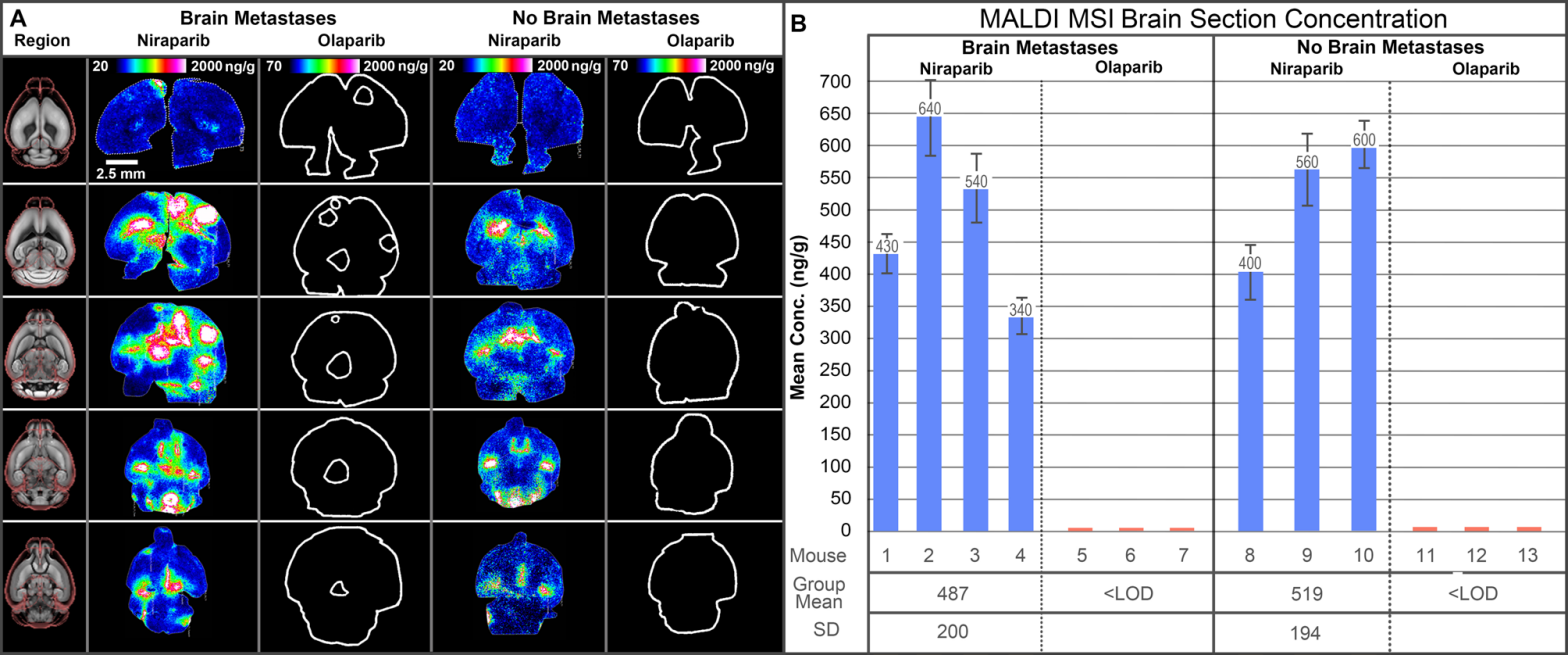
Brain penetration in a mouse model of brain metastases (BM). (**A**) MALDI MSI of representative brain tissue sections collected at five different anatomical planes from BM mice and no-BM mice administered either niraparib or olaparib. White outlines are drawn to indicate the tissue boundaries in sections where no drug signal was detected (for visualization purposes). (**B**) Above: Bar plots displaying mean concentration and standard deviation (error bars) for the concentration of niraparib and olaparib in the BM versus no-BM groups. Below: Table of the mean, standard deviation, and count of the back-calculated concentrations of niraparib and olaparib in the whole brain. BM, brain metastasis; LOD, limit of detection; MALDI MSI, matrix-assisted laser desorption/ionization mass spectrometry imaging.

**Fig. 3:**
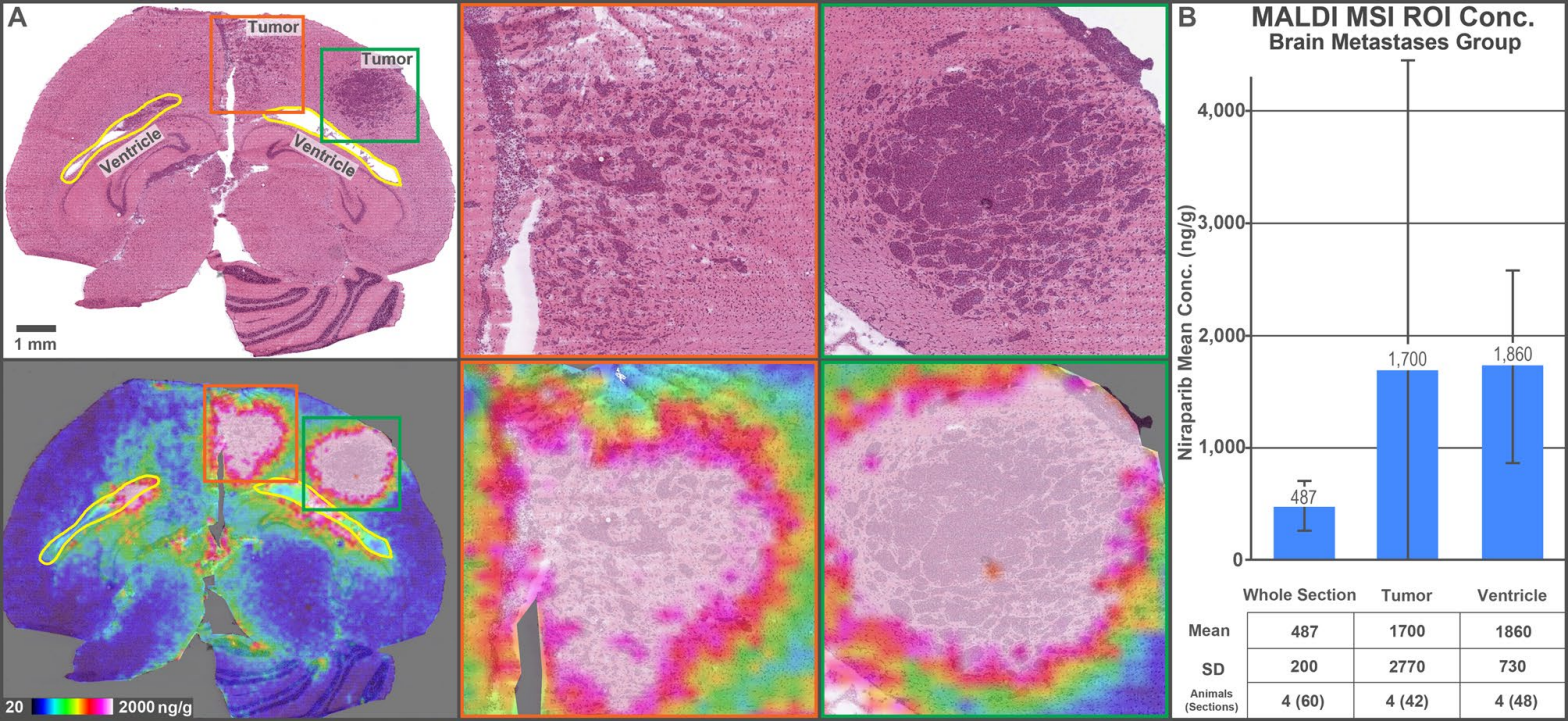
Brain penetration in a mouse model of BM. (**A**) H&E-stained section and MALDI MSI ion image for niraparib generated from a tissue section collected from a mouse with BMs administered niraparib. Magnified view of two tumor regions in the mouse brain. (**B**) Bar plots and table displaying mean concentration and standard deviation (error bars) for the concentration of niraparib in the whole section, tumor, and ventricle ROIs across all sections containing the respective ROI from the BM group treated with niraparib. BM, brain metastasis; Conc., concentration; H&E, hematoxylin and eosin; MALDI MSI, matrix-assisted laser desorption/ionization mass spectrometry imaging; ROI, region of interest.

Ex vivo imaging by MALDI MSI of brain sections from niraparib-dosed mice showed consistent niraparib signal throughout the brain parenchyma across multiple anatomical areas, whereas olaparib was not detected in any examined brain section (Fig. 2). The differences in mean concentration between the niraparib and olaparib treatment groups were statistically significant in both the BM and no-BM mice (*p* < 0.001). The mean brain tissue section concentrations measured for niraparib across all animals by MALDI MSI (519 ng/g no-BM and 487 ng/g BM) were in good agreement with the bulk brain homogenate concentrations measured by LC-MS/MS (658 ng/g no-BM and 543 ng/g BM; Table 2). Similar to in the NHP study, this concordance validates the novel spatial quantification provided by MALDI MSI against the established quantification by conventional bioanalysis. Spatial drug quantification was further interrogated by first co-registering each MALDI MSI brain tissue section with its corresponding H&E-stained section and annotating the major histological features (tumor, ventricles, parenchyma). Quantitative analysis of these regions of interest (ROIs) revealed that niraparib concentrations were highest in the tumor and ventricle regions. A mean concentration of approximately 1700 ng/g was calculated combining the localized tumor concentrations across all tumor-containing sections (*n* = 42) in all BM animals (*n* = 4; Fig. 3). The mean niraparib concentration in the ventricles was approximately 1860 ng/g across all ventricle-containing sections (*n* = 48) in all BM animals. The mean section concentration (including all regions) was approximately 487 ng/g across all sections (*n* = 60) from all BM mice.f The concentrations of niraparib and olaparib were below the LOD for MALDI MSI in the brains of vehicle-dosed mice.

### Brain penetration in an orthotopic glioblastoma model

Finally, the brain penetration and distribution of niraparib and olaparib were measured in an orthotopic GL261 glioblastoma model. Drug concentrations were measured by LC-MS/MS in tumor tissue and in contralateral normal brain tissue at 2 h and 24 h after the last dose (Table 3). Olaparib exhibited very limited distribution to the brain in this model: concentrations in contralateral normal brain at 2 h were ~ 76 ng/g and by 24 h only one of nine mice had detectable olaparib measured at ~ 8 ng/g. In the brain tumor tissue, concentrations at 2 h were ~ 309 ng/g and by 24 h only three of nine mice had detectable olaparib with a mean concentration of ~ 78 ng/g. By contrast, niraparib showed much higher brain exposure in this model. At 2 h post-dose, the mean niraparib concentration in tumor tissue was 7704 ng/g vs. 688 ng/g in contralateral normal brain; at 24 h post-dose, the mean niraparib concentration was 436 ng/g in tumor vs. 171 ng/g in normal brain. Correspondingly, niraparib’s K_p, brain_ was higher in the tumor tissue than in the contralateral normal brain at both timepoints (2 h: K_p, brain_ 0.893 in tumor vs. 0.080 in normal brain; 24 h: 0.465 in tumor vs. 0.182 in normal brain). The K_p, uu, brain_ for olaparib at 2 h (0.031) was slightly higher than that for niraparib (0.011). However, because olaparib’s K_p, brain_ at 2 h was only ~ 0.031 (indicating minimal brain penetration), this comparison is misleading and likely reflects residual cerebral blood. The K_p, uu, brain_ for olaparib at 24 h could not be calculated given that the normal brain concentration was below the quantification limit in eight of nine mice.

**Table 3:** Drug concentrations measured by LC-MS/MS bioanalysis in orthotopic glioblastoma mice after oral administration of niraparib (35 mg/kg) or Olaparib (50 mg/kg) once daily for 3 Days.

	*n*	Time since dose, h	Plasma, mean (SD), ng/mL	Normal brain, mean (SD), ng/g	Brain tumor, mean (SD), ng/g	K_*p*, brain_^a^	K_*p*, uu, brain_^a^	K_*p*, tumor_^b^
Niraparib	9	2	8627 (4863)	688 (305)	7704 (6772)	0.080	0.011	0.893
	9	24	937 (1596)	171 (134)	436 (354)	0.182	0.025	0.465
Olaparib	9	2	2451 (700)	76 (80)	309 (238)	0.031	0.020	0.126
	9	24	23.5 (50.1)	8^c^	78 (92)^d^	NC	NC	NC

C_u, brain, ss_, steady-state unbound drug concentration in brain interstitial fluid; C_u, plasma, ss_, steady-state unbound drug concentration in plasma; F_u_, fraction of unbound drug; K_p, brain_, brain-to-plasma partition coefficient; K_p, tumor,_ tumor-to-plasma partition coefficient; K_p, uu, brain_, unbound brain-to-plasma partition coefficient; LC-MS/MS, liquid chromatography–tandem mass spectrometry; NC, not calculated.

^a^Calculated as ratio of C_u, brain, ss_ (mean normal brain tissue concentration adjusted for F_u_) versus C_u, plasma, ss_ (mean terminal total plasma concentration adjusted for F_u_).

^b^Calculated as ratio of mean brain tumor total concentration versus mean terminal plasma total concentration.

^c^Only one of nine animals had detectable drug levels.

^d^Only three of nine animals had detectable drug levels.

## Discussion

Overall, our results across multiple preclinical models after 3–5 days of oral dosing suggest that niraparib can achieve greater brain penetration than olaparib. Niraparib also achieved localized high concentrations in brain tumors (when present) relative to the surrounding brain regions.

In healthy rhesus macaque NHPs (an intact BBB model), niraparib showed extensive brain penetration, with a mean K_p, brain of_ 3.179 and a mean K_p, uu, brain_ of 0.313. These values reflect niraparib’s ability to readily cross the BBB and distribute throughout the brain. Spatial analysis of brain sections by MALDI MSI provided additional context, estimating a mean unbound niraparib concentration in the brain parenchyma of ~ 15 ng/g - more than twice the predicted pharmacologically relevant concentration of 6 ng/g (19 nM, corresponding to 5x the biochemical IC_50_)^26^. This favorable PK profile in an intact-BBB model supports further investigation of niraparib’s potential preventative effects for protecting against brain metastases.

In the mouse models, the estimated K_p, tumor_ for niraparib approached 1 at 2 h post-dose, suggesting nearly equal drug concentrations in plasma and tumor tissue. The tumor free fractions (F_u_) were not measured and therefore the K_p, uu, tumor_ could not be calculated; nonetheless, the high total niraparib levels achieved in the tumors of both models were noteworthy. Furthermore, in the orthotopic glioblastoma model, niraparib persisted in the tumor region of the brain for 24 h after last dose. These favorable brain tumor PK data in mice are encouraging and warrant further investigation of niraparib in nonclinical efficacy studies and potentially clinical trials as a treatment option for primary and metastatic brain tumors.

Efforts to better understand the mechanism of niraparib’s extensive brain penetration have focused on the role of P-glycoprotein (P-gp; MDR1; ABCB1) and breast cancer resistance protein (BCRP; ABCG2), which are key efflux transporters expressed in the luminal membrane of brain capillary endothelial cells. These transporters are important contributors to the functional integrity of the BBB and regulate brain exposure to drugs that are substrates.

Sun et al. reported in vitro experiments measuring the net efflux ratios of niraparib and olaparib using MDCKII cells overexpressing human P-gp or BCRP transporters^23^. In this work it was shown that both niraparib and olaparib are substrates for P-gp; however, niraparib exhibited significantly lower net efflux ratios, two- to five-fold less than those observed for olaparib^23^. Recent in vivo experiments conducted by Martins et al. using wild-type and genetically modified mice (P-gp and P-gp/BCRP knockouts) also reported that P-gp plays a significant role in modulating the brain exposure of niraparib^29^. Specifically, Martins et al. reported a K_p, brain_ in wild-type mice of 0.20 for niraparib, closely aligning with the K_p, brain_ in the mouse studies reported here (0.19 in brain metastasis model and 0.08 in orthotopic model). The K_p, brain_ in the P-gp knockout mice was 1.20 and further increased to 1.46 in the P-gp/BCRP knockout mice, highlighting the critical role these efflux transporters play in regulating niraparib’s brain concentrations^29^. Notably, niraparib has a high cell membrane permeability^23,30^ and this may play an important role in its ability to overcome transporter-mediated efflux at the BBB and distribute throughout the brain with sustainable exposure.

Niraparib’s K_p, uu, brain_ was significantly higher in NHPs (mean K_p, uu, brain_ of 0.313) compared to mice (brain metastases study: mean K_p, uu, brain_ of 0.026; glioblastoma study: mean K_p, uu, brain_ of 0.011 at 2 h and 0.025 at 24 h). The reason for this interspecies difference is unknown and could be a combination of multiple factors, but it may reflect differences in the expression or activity of BBB efflux transporters between species. It has previously been reported that P-gp protein expression in mice was 3-fold higher than in NHPs and humans, which were similar^31–33^. This expression difference has important translational significance for drugs that are P-gp substrates since it could lead to an overprediction of the impact of P-gp on brain penetration in data generated in mice, thus, the NHP may be a better model to predict the role of P-gp in limiting brain penetration of drugs in humans^34,35^.

The translational relevance of these nonclinical findings is supported by clinical data. In a recent phase 0/2 trial, where a PK ‘trigger’ determined the eligibility for the therapeutic expansion phase, niraparib was administered once daily (200 or 300 mg) for 4 days before surgical tumor resection at either 3–5 or 8–12 h after the last dose^26^. All patients in the phase 0 portion met the PK threshold criteria for the therapeutic phase 2 expansion portion, which required the measured unbound tumor concentrations of niraparib to be greater than 5-fold the biochemical IC_50_ (19 nM). Among 43 patients in the phase 0 cohort, the K_p, tumor_ was reported as 4.2 in nonenhanced tissue and 7.5 in gadolinium-enhanced tissue, indicating that niraparib achieved pharmacologically relevant concentrations in human brain tumors^27^.

One limitation of these studies is the relatively small sample sizes and reliance on a single terminal timepoint for tissue collection in both the NHP and mouse brain metastases models. While tissue collection at steady-state (5 days oral dosing) aimed to mitigate variability associated with PK fluctuations, the use of a single timepoint limits the ability to fully characterize temporal kinetics including region- and time-dependent differences in drug distribution. These constraints must be balanced against resource availability and ethical considerations regarding animal use, especially in higher-order species, where minimizing the number of animals is essential.

It should be noted that the high concentrations of niraparib observed in tumor tissue could be influenced by locally compromised BBB in the tumor microenvironment. The BBB integrity was not directly assessed in the glioblastoma or brain metastasis models, but brain tumors often exhibit leaky vasculature and disrupted BBB regions, which can enhance drug extravasation into tumor tissue^36^. Thus, the increased accumulation in tumor regions may reflect a combination of niraparib’s inherent penetrance and the partial breakdown of the BBB in those areas. Furthermore, characteristics of the tumor microenvironment – such as irregular tumor vasculature, regions of necrosis, and hypoxia – can influence drug penetration and distribution and may lead to heterogeneous drug concentrations within tumors, independent of the drug’s inherent BBB permeability. This heterogeneity in cellular morphology can also impact the localized detection sensitivity in MALDI MSI experiments due to variation in the ionization suppression effects across different regions^37^. To help mitigate the impact of the heterogeneous morphology we incorporated stable label internal standards (niraparib-d7 and olaparib-d8) into the MALDI matrix to enable pixel level normalization of the imaging results; however, these microenvironmental conditions could still have impacted the spatial drug concentration profiles observed in our models.

In summary, these results suggest that niraparib can achieve higher brain penetration than olaparib in the NHP healthy brain and mouse brain tumor models. These data provide evidence that niraparib may offer a better PK exposure profile than olaparib in primary and secondary brain tumors. Moreover, niraparib’s ability to penetrate healthy NHP brain tissue warrant further investigation into the potential for prevention of brain metastasis for some tumor types.

## Methods

### Materials

The luciferase-transfected human breast cancer cell line (MDA231-BrM2-831) was purchased from Memorial Sloan Kettering Cancer Center (New York, NY). Niraparib, deuterated niraparib (niraparib-d7), and olaparib were synthesized internally. Deuterated olaparib (olaparib-d8) was purchased from Alsachim (Illkirch Graffenstaden, France). Cynomolgus macaque brain tissue (lot #CYN252598), rhesus macaque brain tissue (lot #RHS24901), rhesus macaque plasma (lot #RHS24900), CD1 mouse brain tissue (lot #MSE343926), and CD1 mouse plasma (lot #MSE343926) used in the protein-binding assays were obtained from BioIVT (Westbury, NY).

### Animal study conduct

Animal studies were conducted in accordance with the GSK Policy on the Care, Welfare, and Treatment of Laboratory Animals and were reviewed and approved by the Institutional Animal Care and Use Committee at GSK or the external laboratory conducting the study. Animals were housed in a facility accredited by the American Association for Accreditation of Laboratory Animal Care at GSK (Collegeville, PA) or at Charles River Laboratories (Worcester, MA) throughout the study. Animals were appropriately acclimated to handling procedures and restraint devices before study initiation and were handled and housed according to current GSK standard operating procedures. For studies conducted on behalf of GSK at an external laboratory, animals were housed and maintained according to the standard operating procedures of the testing facility. Studies are reported in accordance with the ARRIVE guidelines^38^.

NCr nude mice were group housed (5 per cage) in individually ventilated cages, maintained on a 12:12-hour light: dark cycle, fed a purified mouse diet ad libitum (AIN-93G Rodent Diet, Irradiated; Inotiv, Madison, WI), and had unrestricted access to water from sterile prefilled bottles. The mice were provided with nesting material and huts for structural enrichment. NCr nude mice were single housed for the 5-day oral dosing regimen at the end of the study.

C57BL/6 mice were group housed (5 per cage) in individually ventilated cages, maintained on a 12:12-hour light: dark cycle, fed a standard mouse diet (Teklad Global 18% Protein Rodent Diet, Irradiated; Inotiv) that was supplemented with diet gels and fruit crunchie treats based on needs to encourage eating, and had unrestricted access to water from sterile prefilled bottles. The mice were provided with sterile paper diamond twists and huts for structural enrichment.

Rhesus macaques were housed indoors, maintained on a 12:12-hour light: dark cycle, fed a standard primate diet (Monkey Diet 5038; LabDiet, Richmond, IN) that was supplemented daily with a variety of fresh foods and forage items and had unrestricted access to water. The macaques were provided with toys and auditory or visual enrichment daily.

### Niraparib and Olaparib biodistribution in nonhuman primates

Four healthy male rhesus macaques (*Macaca mulatta*; weight range, 12.0–13.9 kg; Labcorp Drug Development [formerly Covance Research Products], Burlington, NC) were dosed daily via oral gavage for 5 days with either niraparib (*n* = 2; 6 mg/kg) or olaparib (*n* = 2; 10 mg/kg). Animals were randomized using the GSK Randomization App 2.0, which is an internal software tool that uses the ‘sample’ function in R to randomly shuffle animal IDs, then assigns them to treatment groups to ensure unbiased allocation. This study was not statistically powered due to limited availability of NHPs.

Before each dose, animals were lightly sedated with an intramuscular injection of ketamine (5–10 mg/kg), and a blood sample was drawn. On day 5, animals administered niraparib were euthanized at 4 h after dose, and animals administered olaparib were euthanized at 2–2.5 h after dose. NHPs were euthanized by administering an overdose of injected sodium pentobarbital IV (150–200 mg/kg) and confirmed by thoracotomy and exsanguination. Terminal plasma, cerebrospinal fluid, and brain tissue were collected at necropsy. The timing of tissue collection was selected at the approximate plasma time to peak drug concentration (T_max_) for niraparib (≈ 4 h) based on the pharmacokinetic (PK) data available for oral dosing in NHPs (data not reported). For olaparib, because no NHP PK data were publicly available, the timing between the last dose and euthanasia was selected based on the PK data available that estimates T_max_ in humans at 1–3 h after multiple-dose oral administration^39^. The brain was cut into six roughly 1-cm thick coronal segments before snap-freezing by floating on liquid nitrogen. The right hemispheres of the four interior coronal brain segments were cryosectioned and analyzed by matrix-assisted laser desorption/ionization mass spectrometry imaging (MALDI MSI) to quantitatively assess the tissue distribution of the dosed compounds^40–43^. After MALDI MSI analysis, tissue sections were stained with hematoxylin and eosin (H&E) for correlation of ion images with underlying tissue histology. Blood and cerebrospinal fluid (CSF) samples as well as homogenized tissue sections adjacent to those analyzed by MALDI MSI were submitted for liquid chromatography–tandem mass spectrometry (LC-MS/MS) bioanalysis to assess the levels of niraparib and olaparib.

### Niraparib and Olaparib biodistribution in metastatic tumor mouse model

A luciferase-transfected human breast adenocarcinoma cell line (MDA 231-BRM2-831) was used to generate a metastatic brain tumor model in female athymic nude mice. This triple-negative breast cancer cell line was derived from the parental MDA231-TGL cell line after 2 passages in nude mice and is known to specifically metastasize to brain tissue. NCr nude mice (strain: CrTac: NCr-Foxn1^nu^; female; 5–7 weeks old; Taconic Biosciences (Germantown, NY)) were used for these studies. A power analysis was completed by a statistician to determine the appropriate sample sizes for each group to measure a statistically significant. Mice in the metastatic disease group (*n* = 50) received 2.5 × 10^5^ MDA 231-BRM2-831 cells via intracardiac (IC) injection into the left ventricle using a 25-gauge, 5/8-inch-long needle attached to a 1-cc syringe. Control mice (*n* = 10) underwent a sham IC procedure (needle insertion into the ventricle without cell injection. All mice were monitored for tumor development by bioluminescence imaging (BLI) approximately once weekly for ~ 45 days, beginning 1 h after cell injection. In vivo BLI was performed at ~ 10 min post-luciferin injection (corresponding to the peak bioluminescent signal) using an IVIS Spectrum-CT system (PerkinElmer, Waltham, MA). Imaging parameters were set to automatic exposure in the Living Image software, while the binning (8) and aperture (open f-stop) remained at the default settings for all BLI acquisitions. The imaging frequency was increased to twice weekly for mice that developed brain metastases (BMs) as indicated by increased bioluminescence in the cranial region. Only mice that developed clear brain metastases (7 of the 50 injected mice) were included in the treatment phase of the study. The exact tumor size was not measured as an endpoint of this study.

Once BMs were confirmed by BLI, those mice were randomly assigned to a treatment group using the GSK Randomization App 2.0 to receive either niraparib (*n* = 4; 35 mg/kg) or olaparib (*n* = 3; 50 mg/kg) by oral gavage once daily for five days. In parallel, the control (no-cell) mice were administered niraparib (*n* = 3; 35 mg/kg), olaparib (*n* = 3; 50 mg/kg), or vehicle (*n* = 3) by oral gavage once daily for five days. The niraparib dose of 35 mg/kg and olaparib dose of 50 mg/kg were selected based on prior studies to achieve high but tolerable exposure levels in mice, with olaparib’s higher dose reflecting its lower bioavailability. Two hours after the final dose, mice were euthanized by CO_2_ asphyxiation. At necropsy, a terminal blood sample was collected via cardia puncture, and the brains were harvested and snap-frozen by floating on liquid nitrogen.

Cryosections of brain tissue were collected from 5 distinct horizontal planes across all animals. From each plane, serial tissue sections were collected for MALDI MSI and for H&E staining, and immunohistochemistry (IHC) staining. Tissue trimmings from between each plane were collected, homogenized, and submitted for bioanalysis in addition to the terminal plasma samples.

### Niraparib and Olaparib biodistribution in an orthotopic glioblastoma (GL261) model

Female C57BL/6 mice were inoculated intracranially on the right side of the brain in the right frontal lobe with mouse glioblastoma (GL261) tumor cells (≈ 2 × 10^5^) in 3 µL of phosphate-buffered saline for the tumor development. Mice were observed daily for clinical signs, and any mouse meeting predefined criteria for moribund status was euthanized. Criteria for moribund sacrifice included: ≥ 20% body weight loss or severe neurological symptoms (e.g., prostration, paralysis, seizures, hemorrhage). Of the 72 tumor-inoculated mice, 13 were euthanized early as moribund due to weight loss > 20% and/or neurological symptoms, and 1 mouse was found dead prior to study completion. The dosing regimens used in this study have previously been established as tolerable in mice and therefore the observed toxicity was primarily driven by brain tumor progression.

Intracranial tumor growth was not monitored during the in-life phase of the study but prior experience with the GL261 model’s growth kinetics showed that tumors are typically established by roughly two weeks after inoculation. Thus, thirteen days after tumor inoculation, mice were randomized by animal ID number into treatment groups to receive either niraparib (35 mg/kg; 2 h (*n* = 9) and 24 h (*n* = 9) timepoints) or olaparib (50 mg/kg; 2 h (*n* = 9) and 24 h (*n* = 9) timepoints) via oral gavage once daily for 3 days. (The remaining inoculated mice were allocated to other treatment groups receiving a different compound and are not included in this report.) The original study design aimed at 5 days of oral administration; however, many mice were showing body weight loss of > 20% and moribund status; therefore, the collection day was changed to day 3 instead of day 5. Mice were euthanized by CO_2_ asphyxiation. At necropsy, terminal blood, brain tumor tissue, and contralateral normal brain tissue were collected from each animal at 2–24 h after the final dose on day 3 for both niraparib and olaparib groups. Plasma and tissue samples were analyzed by LC-MS/MS to measure niraparib and olaparib concentrations. MALDI MSI was not conducted in this study because the tumor tissue could be effectively isolated at necropsy from the contralateral normal brain and therefore the LC-MS/MS bioanalysis was prioritized to maximize detection sensitivity.

### Matrix-assisted laser desorption/ionization mass spectrometry imaging (MALDI MSI)

For both the nonhuman primate (NHP) and mouse metastases model studies, brain tissue sections of 6-µm thickness were prepared on a Leica CM1950 cryostat (Leica Biosystems, Danvers, MA). For the NHP study, tissue sections were collected from four of the coronal brain segments from each animal. From each coronal segment, three tissue sections were collected for MALDI MSI, one section for hematoxylin and eosin (H&E) staining, and roughly 30 sections were collected into a pre-weighed vial for homogenization and liquid chromatography–tandem mass spectrometry (LC-MS/MS) bioanalysis. The three sections for MALDI MSI were thaw-mounted onto double-width indium tin oxide–coated slides (BigSlides; Bruker Daltonics, Billerica, MA) that had been pretreated with a poly-l-lysine solution to improve tissue adherence. The section for H&E staining was thaw-mounted onto a standard double-width glass microscope slide.

For the mouse metastases study, tissue sections were collected from five horizontal tissue planes starting from roughly 1 mm from the top of the tissue, and each subsequent tissue plane was separated by roughly 1 mm. At each tissue plane, three tissue sections were collected for MALDI MSI, one section for H&E staining, and one to five sections for immunohistochemistry (IHC) staining (five sections from animals that had brain metastases and one section from animals in the control group that did not receive a cell injection). For MALDI MSI analysis, sections were thaw-mounted onto indium tin oxide–coated slides (Delta Technologies, Ltd, Loveland, CO). For H&E and IHC analyses, tissue sections were collected onto superfrost glass slides (Fisher Scientific, Waltham, MA). Brain tissue trimmings from between each of the horizontal planes were pooled and collected into a pre-weighed vial for homogenization and LC-MS/MS bioanalysis (described in detail later).

To perform quantitative MALDI MSI, a mimetic tissue model was constructed for niraparib and olaparib using a procedure modified from that previously published^42,43^. A gelatin cast of the mimetic model was prepared by pipetting a warmed (50 °C) 15% gelatin solution into a tissue microarray mold (Arraymold kit C, 4 mm, 15 cores; Arraymold, Ogden, UT). The gelatin cast was then frozen overnight at (− 20 °C). Bulk homogenate of control rat brain tissue was prepared using an Omni Bead Ruptor Elite bead mill homogenizer (Omni International, Kennesaw, GA). The rat brain homogenate was separated to 12 aliquots (≈ 400 mg each), which were spiked with small volumes (< 2% wt/wt) of niraparib or olaparib standard solutions to give an inverse range of tissue concentrations (0.1, 0.2, 0.3, 0.5, 1, 5, 10, 25, 50, 75, and 100 µg/g, as well as a blank homogenate) for niraparib (0.1–100 µg/g) and olaparib (100–0.1 µg/g). The gelatin cast was warmed to room temperature, and the spiked homogenates were pipetted into each of the individual wells. The cast and homogenate were then frozen by floating on liquid nitrogen and stored at − 80 °C. Cryosections (6-µm thickness) of the mimetic tissue model were then collected on the same slide as the sample tissue to be quantified.

Slides for MSI were coated with a matrix solution consisting of 25-mg/mL 2,5-dihydroxybenzoic acid dissolved in methanol: water: DMSO: TFA (50:50:0.5:0.1) and also contained 0.1 µg/mL of internal standard (either deuterated niraparib [niraparib-d7] or deuterated olaparib [olaparib-d8]). Slides were coated with a TM Sprayer (HTX Technologies, Chapel Hill, NC) using the following parameters: 0.1-mL/min flowrate, 70 °C, 3-mm track spacing with CC pattern, and 8 passes. This process results in a tissue concentration of the internal standard of roughly 3 µg/g.

MALDI MSI was performed on either a Bruker ScimaX (7T) or a Bruker SolariX (7T) FT-ICR mass spectrometer. Data were acquired at a spatial resolution of 75 μm in positive polarity using narrowband (65k data size with a 40 m/z window) and continuous accumulation of selected ion (CASI; 15 m/z window) methods that were optimized for either niraparib or olaparib. Both narrowband and CASI windows were centered between parent and internal standard m/z. The small laser focus was used with 200 laser shots per position, and the laser power was adjusted in accordance with a quality control sample acquired just before initiating the imaging sequence.

After MALDI MSI acquisition, slides were immersed in 100% ethanol for 5 min to remove the MALDI matrix. Slides were then stained with H&E and digitally scanned. Ion images were loaded into flexImaging (Bruker Daltonics), the post–MALDI H&E image was coregistered, and key regions of interest (ROIs) for the tissue and mimetic model were drawn. Ion intensities from these ROIs were exported using a 2-ppm window for all relevant ions (niraparib, niraparib-d7, olaparib, and olaparib-d8). A pixel-by-pixel normalization to the internal standard was performed if the whole-tissue ROI was deemed to be significantly different from the blank ROI in the mimetic model. This stipulation was implemented to avoid internal standard normalization of noise, which can introduce artifactual distributions. The intensity ratio of analyte to internal standard from each level of the mimetic model was used to build a standard curve to convert the ion intensity in the tissue ROIs into concentration. All ion images shown are scaled from the limit of blank to 2 µg/g of drug. All quantification calculations were completed using the methods and spreadsheet workflow described by Barry et al.^43^. Data tables capturing the mean concentration and standard deviation from each dataset were generated and imported into Spotfire (TIBCO, Palo Alto, CA) to create the bar chart figures in the main text. To assess differences between compounds, an unpaired, two-tailed Welch’s t-test was performed using individual section concentrations as replicates. Statistical significance was set at *p* < 0.05.

Supplementary statistical analyses were conducted on the MALDI MSI data for both the murine brain metastasis and NHP brain studies by fitting a mixed-effects model with the response variable log10(concentration [in ng/g] + 0.001) and an animal/plane as nested random effects. For the mouse brain metastasis model, we included the following fixed effects: (1) treatment, (2) region, (3) metastasis group, and (4) all two-way and three-way interactions of (1) to (3). For the NHP model, we included the following as fixed-effects: (1) treatment, (2) region, and (3) two-way interaction of (1) and (2). The median concentration from each ROI was used in these analyses. All statistical analyses were conducted in R version 4.2.1(R Foundation for Statistical Computing, Vienna, Austria)^44^. Linear mixed-effects models were fit using the lme4 and lmerTest packages, and estimated marginal means and CIs were derived using the emmeans package^45–47^. All figures from these analyses were created using the ggplot2 package and are included in the Supplemental Data as Figures S3 and S4^48^.

### LC-MS/MS bioanalysis methods for NHP and mouse BM studies

Brain tissue was weighed in polypropylene tubes, then phosphate-buffered saline (PBS) was added to the tissue sample before homogenization (1:4 wt: vol). Matrix-matched standard curves were prepared for plasma, brain homogenate, and CSF (NHP study only) samples, then samples were analyzed for niraparib or olaparib concentration using specific and sensitive high-performance LC-MS/MS methods using protein precipitation with acetonitrile containing an internal standard.

The LC-MS/MS system includes an API-6500 triple quadrupole mass spectrometer (Sciex, Framingham, MA) and an ExionLC system (ExionLC Controller, ExionLC AD Column Oven, ExionLC AD Pump, and ExionLC AD Multiplate Sampler; all from Sciex). Data were acquired and processed using Analyst^®^ 1.7.2 software (Sciex). An Acquity UHPLC BEH C18 analytical column (1.7-µm, 2.1 × 50 mm; Waters, Milford, MA) was applied in sample analysis.

For niraparib, the LC-MS/MS method lowest limit of quantification (LLQ) was 2.00 ng/mL and highest limit of quantification (HLQ) was 10,000 ng/mL for all matrices (plasma, brain homogenates, and CSF). For olaparib, the LLQ was 1.00 ng/mL and HLQ was 10,000 ng/mL for all matrices (plasma, brain homogenates, and CSF).

### LC-MS/MS bioanalysis methods for orthotopic glioblastoma mouse model study

Brain tissue was weighed in polypropylene tubes, then PBS was added to the tissue sample before homogenization (1:4 wt: vol). Matrix-matched standard curves were prepared for plasma and brain homogenate, then samples were analyzed for niraparib or olaparib concentration using specific and sensitive LC-MS/MS methods using protein precipitation with acetonitrile containing an internal standard.

The LC-MS/MS system included an API-6500 triple quadrupole mass spectrometer (Sciex). Data were acquired and processed using Analyst^®^ 1.7.2 software (Sciex). An Acquity UHPLC BEH C18 analytical column (1.7-µm, 2.1 × 50 mm; Waters) was applied in sample analysis.

For niraparib, the method had LLQ of 5.00 ng/mL and HLQ of 1000 ng/mL. For olaparib, the LLQ was 5.00 ng/mL, and the HLQ was 1000 ng/mL.

### In vitro determination of the plasma and tissue protein binding

The unbound fractions (F_u_) for niraparib and olaparib were determined by rapid equilibrium dialysis at a nominal concentration of 1 µM, using a dialysis buffer of PBS (100 mM sodium phosphate + 150 mM sodium chloride, pH 7.4). Brain (diluted 1:4 [5×] in PBS) or plasma (undiluted) were spiked with each test compound and applied to each matrix. Aliquots of each mixture were added to the donor wells of a rapid equilibrium dialysis plate in triplicate, and blank PBS was added to each receiver well, at volumes recommended by the manufacturer. Plates were incubated with shaking at 200 rpm for 4 h at 37 °C. At the end of incubation, samples were collected from each donor and receiver well. Samples were matrix-matched with the addition of an equivalent volume of blank matrix to each receiver sample and blank PBS to each donor sample. Additional samples were collected from each incubation mixture at the beginning (T = 0 min) and end (T = 240 min) of incubation for determination of stability, and matrix-matched with PBS as described above. The resultant samples were extracted by protein precipitation with acetonitrile containing an analytical internal standard, centrifuged, and analyzed by LC-MS/MS.

### Cytochrome C oxidase subunit 4 (COXIV) IHC methods—mouse BM study

Mouse brain sections were IHC stained with the rabbit (Rb) monoclonal primary antibody for COXIV (3E11;cat #4850; Cell Signaling Technology, Danvers, MA) on the Roche Ventana Discovery Ultra automated staining platform using the Discovery ChromoMap 3,3′-diaminobenzidine (DAB) detection kit (cat #05266645001; Roche Diagnostics, Indianapolis, IN). Briefly, the COX IV IHC assay was optimized and performed with the following steps. Slide sections were baked and deparaffinized onboard the instrument. Afterwards, any nonspecific staining was blocked with normal goat serum. Heat-induced epitope retrieval was performed with CC1 Tris-based buffer for 40 min, followed by COXIV primary antibody incubation for 32 min (per the automated stainer’s protocol). Endogenous peroxidase activity was blocked, and nonspecific binding of secondary antibody was blocked with Antibody Block (cat #05268869001; Roche Diagnostics), followed by the application of OmniMap anti-Rb horseradish peroxidase secondary antibody (cat #05269679001; Roche Diagnostics). After washing, slides were stained with DAB chromogen and subsequently counterstained with hematoxylin and then treated with Ventana HE 600 Bluing Reagent (Roche Diagnostics) to blue the hematoxylin. Whole-slide scanning was performed using a Hamamatsu NanoZoomer S210 digital slide scanner (Hamamatsu Photonics, Shizuoka, Japan).

### Brain and plasma K_p_ and K_p, Uu_

To characterize brain distribution of niraparib and olaparib in the three animal models described above, we calculated the unbound brain tissue partition coefficient (K_p, uu, brain_), which describes the balance of drug influx and efflux across the BBB^7^. First, the brain tissue partition coefficient K_p, brain_ was calculated using Eq. (1).

1 $${\text{K}}_{{{\text{p}},{\text{brain}}}} = {\text{C}}_{{{\text{ss}},{\text{brain}}}} /{\text{C}}_{{{\text{ss}},{\text{plasma}}}}$$

Where C_ss, brain_ is the mean steady-state total drug concentration in brain tissue, and C_ss, plasma_ is the mean steady-state total drug concentration in plasma (both measured by LC-MS/MS). The free (unbound) fractions (F_u_) of niraparib and olaparib in brain and plasma (for both NHPs and mice) were measured in vitro using rapid equilibrium dialysis protein binding assays (Table S1). Finally, K_p, uu, brain_ was calculated using Eq. (2).

2 $${\text{K}}_{{{\text{p}},{\text{uu}},{\text{brain}}}} = {\text{ K}}_{{{\text{p}},{\text{brain}}}} \times {\text{ }}\left( {{\text{F}}_{{{\text{u}},{\text{brain}}}} /{\text{F}}_{{{\text{u}},{\text{plasma}}}} } \right)$$

## Supplementary Information

Below is the link to the electronic supplementary material.

Supplementary Material 1

## Data Availability

The data presented in this study are available on request from the corresponding author. The data are not publicly available due to intellectual property rights.
