# Supplementary material for: Assessment of brain penetration and tumor accumulation of niraparib and olaparib: insights from multimodal imaging in preclinical models
Source: Sci Rep. 2025 Sep 29;15:33670. doi: 10.1038/s41598-025-18255-9 (PMC12480552; doi:10.1038/s41598-025-18255-9)
Supplement: Supplementary file 1 — Supplementary Material 1 [file 41598_2025_18255_MOESM1_ESM.docx]

**Supplementary Information**

**Table S1.**

Niraparib and Olaparib Protein Binding in Different Matrices

| **Analyte** | **Species** | **Matrix** | **F_u_, %** |
| --- | --- | --- | --- |
| Niraparib | Cynomolgus macaque | Brain | 3.61 |
| Niraparib | CD1 mouse | Brain | 2.95 |
| Niraparib | Rhesus macaque | Plasma | 36.6 |
| Niraparib | CD1 mouse | Plasma | 22.0 |
| Olaparib | Rhesus macaque | Brain | 19.4 |
| Olaparib | CD1 mouse | Brain | 23.0 |
| Olaparib | Rhesus macaque | Plasma | 30.1 |
| Olaparib | CD1 mouse | Plasma | 35.2 |

F_u_, fraction of unbound drug.

**Figure S1.**


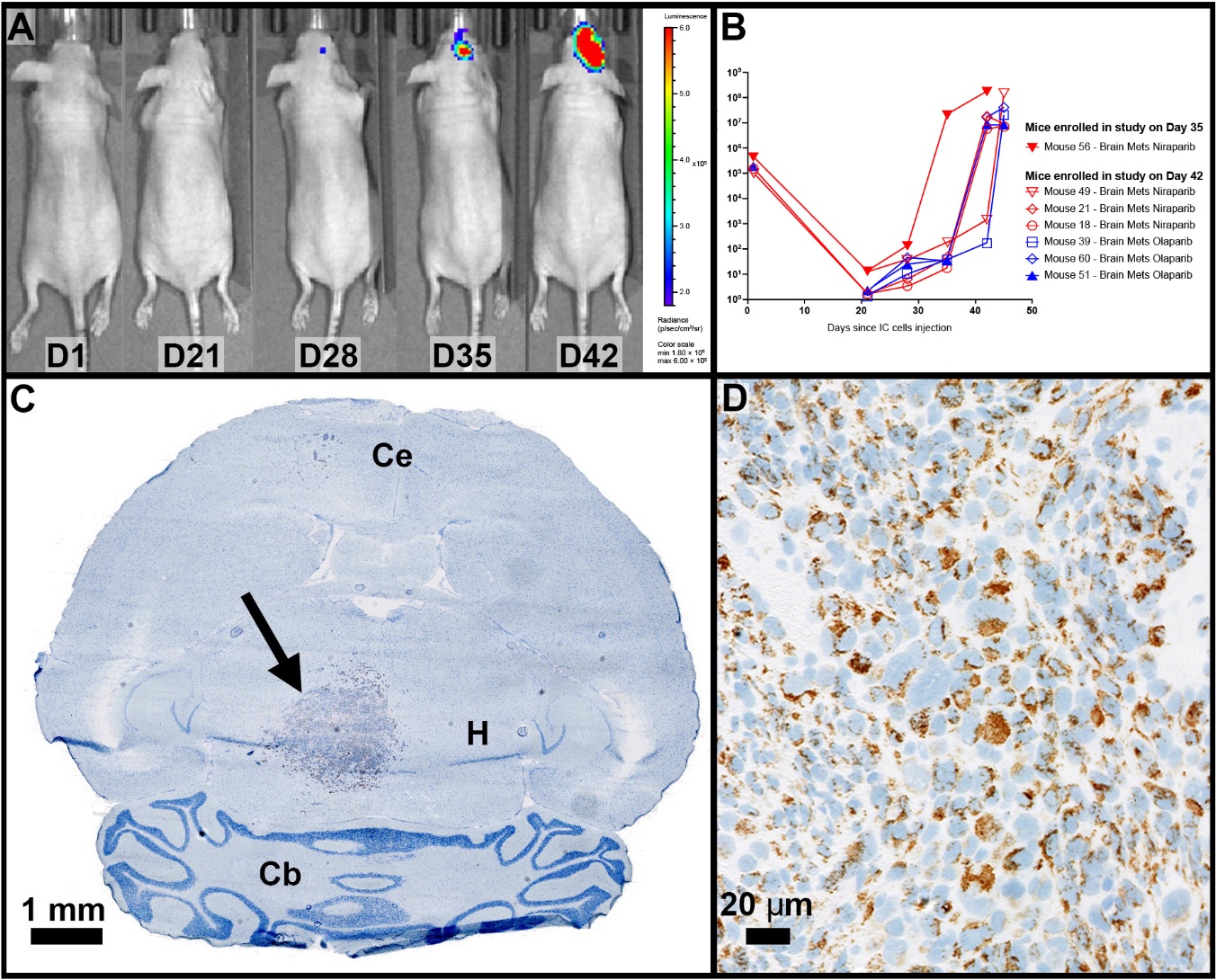


In vivo BLI. (**A**) Representative twice-weekly BLI of a mouse that developed a BM after injection of luciferase-transfected human breast cancer cells (MDA 231-BrM2-831). (**B**) Summary of total flux generated by BLI in mice that developed a BM. (**C**) Microscopic IHC-stained horizontal section of brain, after application of anti-COXIV antibody and hematoxylin counterstain. A solitary cerebral mass is present (arrow) within the cerebrum that includes portions of the hippocampus (rostral to the cerebellum). (**D**) Magnified view of cerebral mass. IHC DAB-positive staining cells (brown) within the mass are readily evident and verify non-mouse derivation of cell populations comprising the mass in the mouse model. BLI, bioluminescence imaging; BM, brain metastasis; COXIV, cytochrome c oxidase subunit 4; D, day; DAB, 3,3′-diaminobenzidine; IC, intracardiac; IHC, immunohistochemistry; Max, maximum; mets, metastases; Min, minimum.

**Figure S2.**


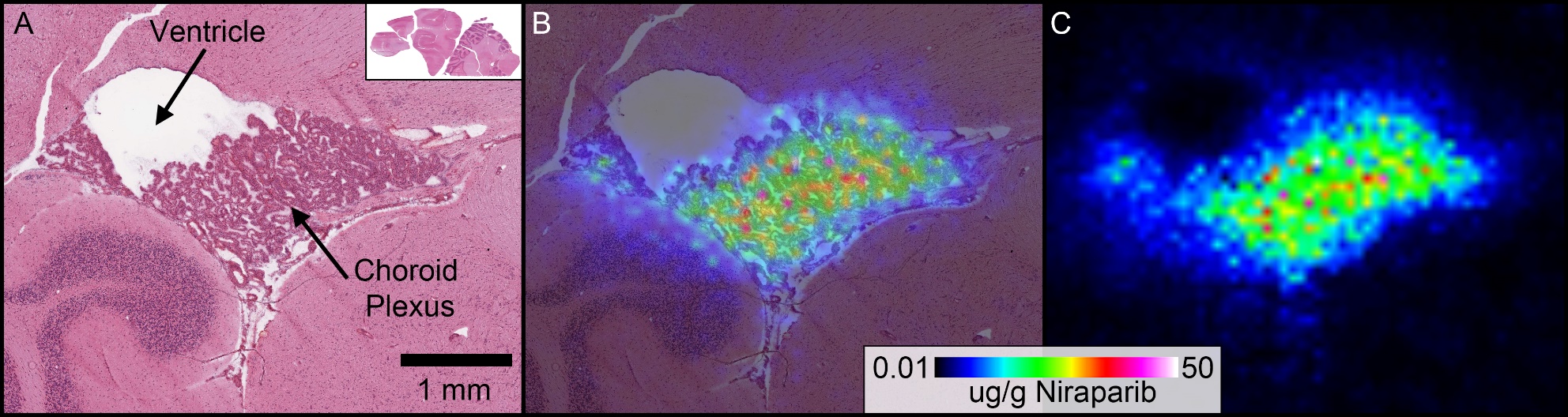


MALDI MSI of Niraparib in NHP Brain A) Magnified view of a ventricle region from a representative NHP brain tissue section stained with H&E. B) Overlay of the co-registered niraparib ion image and serial H&E C) Niraparib ion image

**Figure S3.**


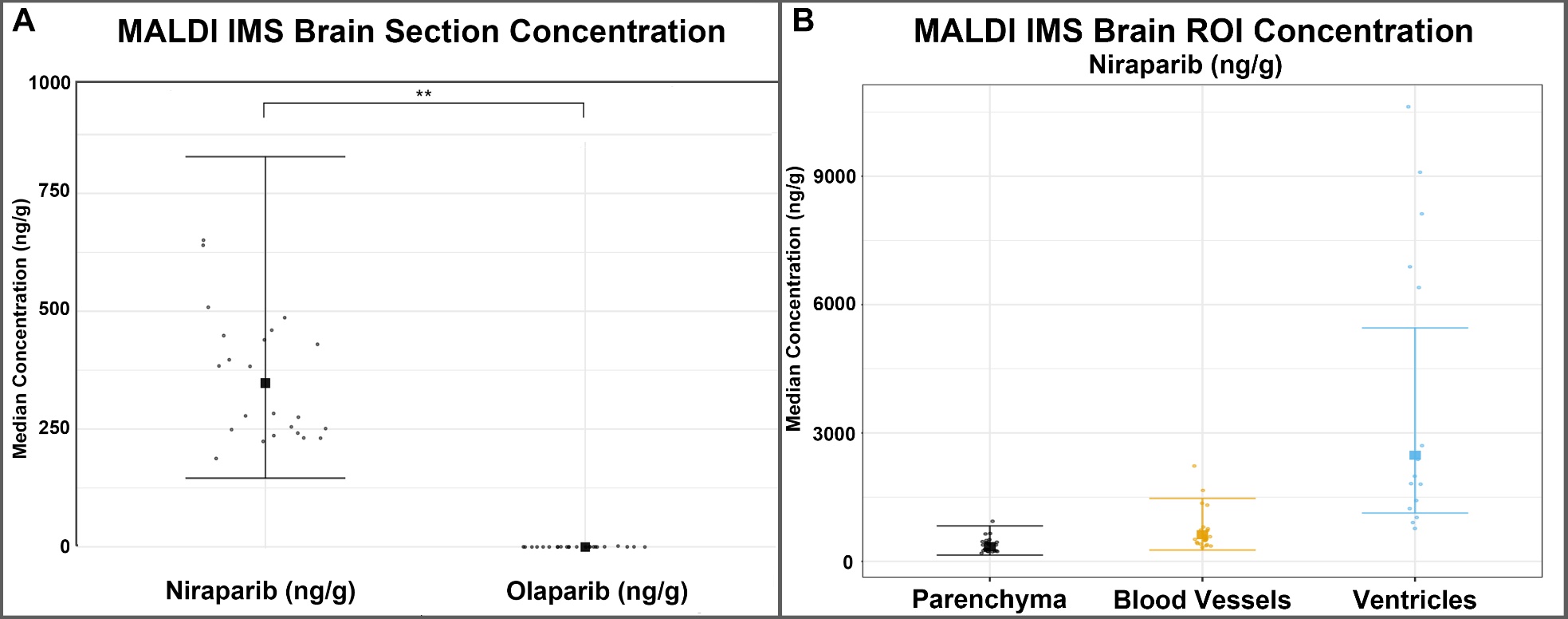


MALDI MSI of niraparib and olaparib in NHP brain (**A**) Tissue section median concentration box plots for niraparib and olaparib dosed NHPs. ***P* < .001. **(B)** Estimated marginal means of the concentration of niraparib in each of the ROIs (square points) with 95% CIs of these estimated marginal means (error bars). This model estimates concentration and accounts for the replicate-to-replicate variability.

**Figure S4.**


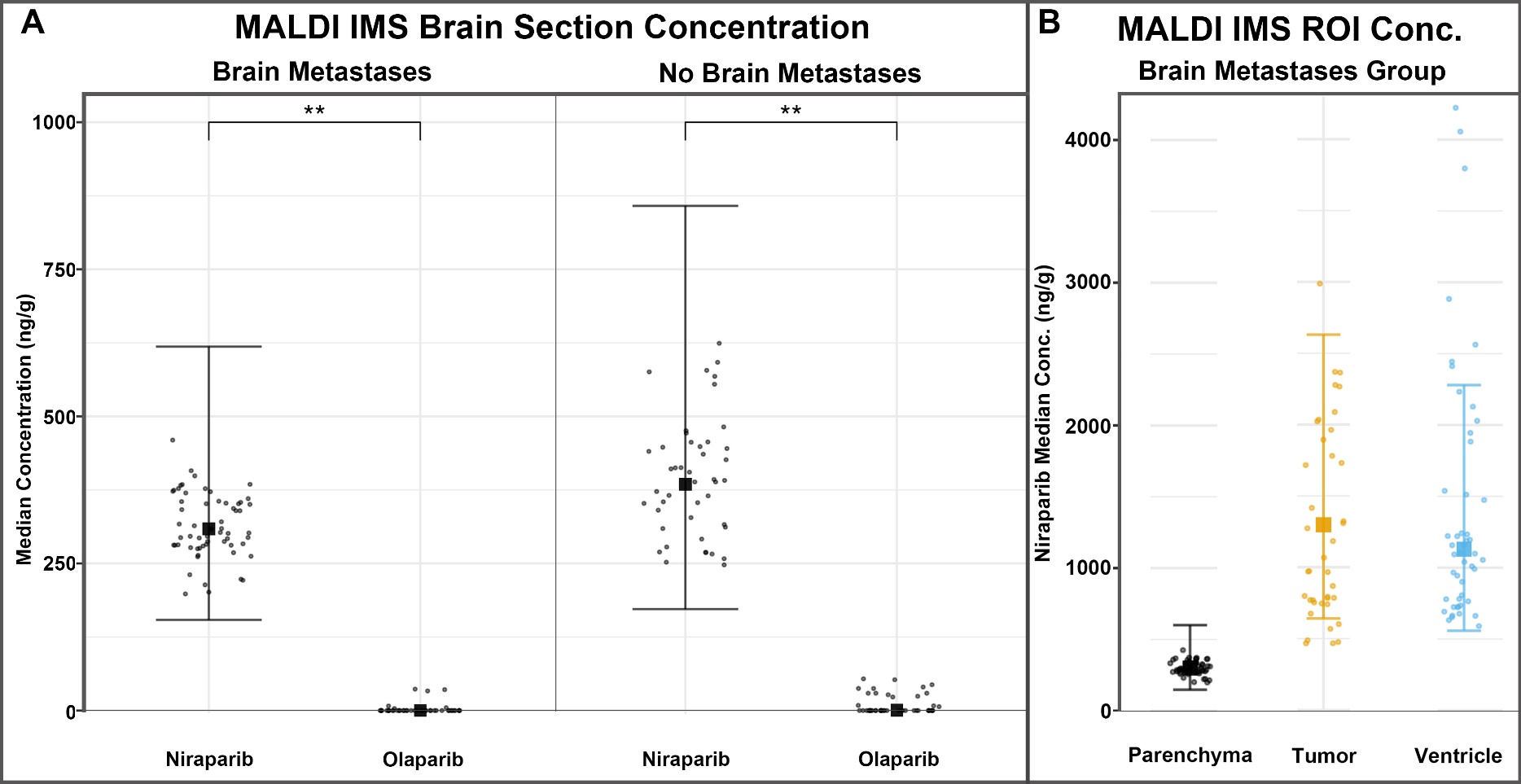


MALDI MSI of niraparib and olaparib in mouse brain metastases model (**A**) Estimated marginal means (square points) and 95% CIs of the estimated marginal means (error bars) for the concentration of niraparib and olaparib in the BM versus no-BM groups. ***P* < .001. **(B)** Estimated marginal means (square points) and 95% CIs of the estimated marginal means (error bars) for the concentration of niraparib in regions of interest in the BM group.
